# Prevalence and Individual-Level Determinants of Uptake of Three or More Doses of Sulphadoxine-Pyrimethamine for Intermittent Preventive Treatment of Malaria in Pregnancy in Busia County, Kenya

**DOI:** 10.4236/ojepi.2024.14302

**Published:** 2024-06-29

**Authors:** Anne Nduta Miatu, Betsy Rono Cheriro, Kamija Samuel Phiri

**Affiliations:** 1 School of Public Health, Department of Environmental Health and Disease Control, Jomo Kenyatta University of Agriculture and Technology, Nairobi, Kenya; 2 School of Global and Public Health, Kamuzu University of Health Sciences, Blantyre, Malawi

**Keywords:** Malaria in Pregnancy, Antenatal Clinic, Intermittent Prevention of Malaria in Pregnancy, Dosage, Uptake, Sulphadoxine-Pyrimethamine and Individual Level Factors

## Abstract

**Background:**

Malaria in pregnancy causes maternal anemia, low birth weight, intrauterine growth retardation, and preterm deliveries. In malaria-endemic regions in Kenya, percentage of pregnant women hospitalized with malaria reach up to 60%. WHO recommends at least three doses of sulphadoxine pyrimethamine for Intermittent Preventive Treatment of Malaria in Pregnancy (IPTp) antenatally. This study sought to ascertain the prevalence and individual-level factors influencing the uptake of IPTp-SP3+.

**Methods:**

A facility-based cross-sectional study at Busia County Referral Hospital. 384 mothers were consecutively sampled at the maternity unit during delivery. Semi-structured questionnaires were used to collect data. Odds ratio (OR) and adjusted OR were used to determine statistical significance of individual factors influencing uptake of three or more IPTp-SP.

**Results:**

43.0% of participants took IPTp-SP3+. Individual factors that affected the uptake of IPTp-SP3+ included starting ANC visits in the first trimester (adjusted odds ratio (aOR) = 2.1, 95% CI: 1.23 – 3.67, p = 0.046), having more than four ANC visits (aOR = 3.1, 95% CI: 1.49 – 6.50, p = 0.002), having a higher monthly income (aOR = 2.6, 95% CI: 1.24 – 5.36, p = 0.012), being aware of the advantages of IPTp-SP medications (aOR = 3.7, 95% CI: 1.40 – 9.74, p = 0.008), and having a positive attitude toward ANC services (aOR = 3.2, 95% CI: 1.61 – 6.31, p = 0.001).

**Conclusion:**

Less than half of the pregnant mothers are complyingIPTp-SP3+. There should be aggressive efforts by the County and National Ministries of Health promoting initiation of ANC attendance early and attendance of all the recommended eight visits together with ensuring availability of the drugs.

## 1 Background

Malaria remains a health problem in tropical and subtropical countries despite significant worldwide efforts to eliminate it, with Sub-Saharan Africa accounting for most deaths. Approximately 50% of the world’s population is at risk from malaria. The Global Malaria Report [1] projected 241 million cases of malaria worldwide in 2020, originating from 85 countries where malaria is endemic. This prevalence increased dramatically from 227 million cases reported in 2019, with most of the increase occurring in Africa [1]. Over that time, 627,000 fatalities were estimated to have occurred. Infections increase (from 213 million to 228 million) and deaths (from 534,000 to 602,000) were predicted to have occurred in Africa, attributing to 95% and 96% of global cases and fatalities, respectively [1]. Although the danger of malaria is not evenly dispersed, it is believed that 70% of Kenyans are susceptible to malaria. Areas most at risk of malaria infection are those around Lake Victoria and the coast. The country-wide prevalence rate is 8%, with the highest incidence (28%) seen in the Lake Endemic area [2]. Busia County, Kenya, is part of the Lake Endemic Zone.

Pregnant women and young children are more vulnerable to malaria infection and mortality. Malaria infection in pregnancy (MiP) carries a number of major risks and problems, including maternal anemia, low birth weight (LBW) babies, intrauterine growth retardation (IGR), early deliveries, and baby mortality during gestation [3]. Inadequate nutrition of the fetus due to malaria parasites can result in low birth weight, a significant contributor to low infant growth and survival in Africa. Low malaria-related birth weight in endemic areas is estimated to be responsible for 6% of newborn deaths [4]. An estimated 11.6 million pregnant women in 33 African countries were exposed to malaria in 2020, resulting in 819,000 low-birth- weight babies [1]. MiP prevention lowers the risk of low birthweight by 43%, perinatal deaths by 27%, and severe maternal anemia by 38%. In Kenya, Ministry of Health (MOH) found that hospitalized malaria cases among pregnant women ranged from 1% to 60% in 2019 [5]. MOH also estimated that 6.3% of women who made their first antenatal care (ANC) visit were hospitalized due to malaria [6].

Sulfadoxine-pyrimethamine (SP) intermittent preventive treatment during pregnancy (IPTp) is a safe and economical way to reduce the burden of MiP and adverse pregnancy and birth outcomes [5]. The World Malaria Report [1] states that 32% of all pregnant women in 33 countries received at least three doses of IPTp. According to this report, if ninety percent of all pregnant women would receive IPTp3, 206,000 low birth weights would be prevented [1]. In 2012, the World Health Organization (WHO) revised its guidelines for administering IPTp and recommended administering SP in three doses or more (IPTp-SP+3). Evidence show that there would be a major global reduction in the number of low birthweight babies if IPTp-SP+3 uptake occurred in at least 90% of all pregnant women [1]. Despite the Kenyan government adopting these policies in 2013 and rapid improvements expected progress in IPTp-SP+3 uptake has been extremely slow. In 2020, only 49% and 46% of Kenyan women aged 15 - 49 in the Lake endemic region and Coast endemic zones received IPTp-SP+3 [7].

Unfortunately, few studies examining the prevalence and factors influencing IPTp-SP+3 uptake been carried out in Kenya, particularly in the endemic lake region. The purpose of this study was to ascertain the prevalence and factors associated with IPTp-SP+3 of MiP. The specific study objective was to determine the prevalence and individual-level factors influencing IPTp-SP+3 of MiP at Busia County Referral Hospital, Kenya.

## 2 Methods

### 2.1 Aim, Design, and Setting

The study aimed to determine the prevalence and individual-level factors affecting IPTp-SP3+ uptake among MiP. The study was a facility-based cross-sectional study, chosen because it is suitable for collecting data at a particular point in time. The study setting was a referral hospital in Busia County, Kenya. It is an appropriate setting considering that 157,650 women of reproductive age are at risk of malaria infection in Busia County [8].

### 2.2 Participant Characteristics, Sample, and Sample Size

Mothers who gave birth at the facility’s maternity unit or those who had recently given birth at home and presented themselves to the facility within 24 hrs were the study participants. The inclusion criteria included mothers with term deliveries (>37 weeks), mothers in possession of their ANC booklet, and mothers visiting the Busia County referral hospital for delivery or who had just delivered at home (within 24 hours before contact) and presented themselves to the facility. Mothers who received treatment for malaria during their pregnancy because of the treatment regimen which is different from the SP, mothers allergic to sulfur, mothers with HIV who were taking daily prophylactic cotrimoxazole, mothers who were in distress and unable to finish the study, and mothers whose preterm deliveries were less than 37 weeks were excluded from the study. Mothers were approached and informed about the study after checking themselves into the facility’s maternity ward, were scheduled for delivery, and had term delivery. Mothers who met the inclusion criteria and consented to participate in the studyby signing the informed consent form were recruited in the study through the consecutive sampling method until the estimated sample size was reached. A sample size of 384 participants had been calculated using the Cochran’s formula [9] with a prevalence of uptake of 49% [10].

### 2.3 Data Collection Tool and Procedures

Semi-structured questionnaires were used to collect data from the study participants. The number of IPTp-SP3+ doses received during pregnancy as well as individual-level factors—such as mother’s age, educational attainment, marital status, and occupation—that affect IPTp-SP3+ uptake were recorded in the questionnaire. Data was collected from June 7, 2023, to July 2, 2023. Two research assistants were enlisted to assist with data collection under the supervision of the principal investigator. The two assistants underwent a two-day training on the study protocol prior to the data collection. They administered the questionnaire to participants and entered the data into Microsoft Excel. The principal investigator compiled, validated, cleaned, and assessed the data for accuracy and completeness.

### 2.4 Data Analysis

Descriptive statistics were used to report on frequencies, percentages, and measures of central tendency. The factors influencing IPTp-SP3+ uptake were evaluated using logistic regression model, presented as Crude Odds Ratio (OR) and Adjusted OR to determine statistical significance (p value) at a 95% confidence interval (CI). A p value of <0.05 was considered for inferential statistics analysis. All analyses were done in SPSS version 25.

## 3 Results

### 3.1 Baseline Sociodemographic Characteristics

The mean age of the participants was 26.51 ± 6.1 years with majority (33.6%) aged between 20-25 years. Most of them were married (79.9%), had secondary education (43.8%), were unemployed (52.9%), resided in rural areas (92.2%), and earned less than KES 10,000 a month (87.5%) (Table 1).

### 3.2 Baseline Obstetric Characteristics

Most participants had 1-2 previous children (62.5%), began their antenatal clinic in the second trimester (50.8%), had four antenatal clinic visits (26.8%), and sought ANC services from a public hospital (95.8%) (Table 2).

### 3.3 Prevalence of Uptake of Three or More Doses of IPTp-SP

**Figure 1:**
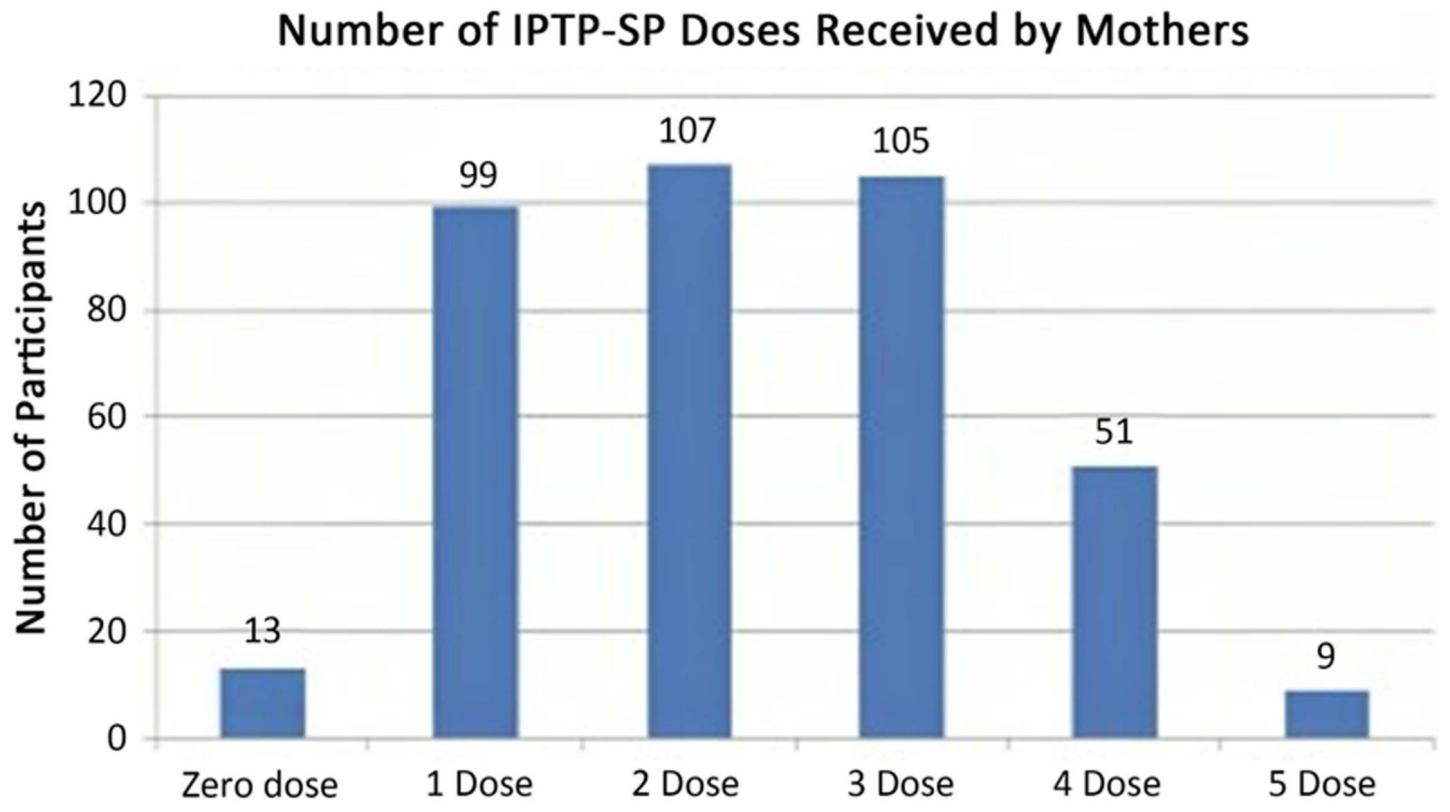
The number of IPTp-SP doses participants received. IPTP-SP intermittent preventive treatment during pregnancy with sulfadoxine-pyrimethamine.

Majority (27.9%) took two IPTp-SP doses during their pregnancy period. 25.8% took one dose, 27.3% took three doses, 13.3% took four doses, 2.3% took 5 doses, and 3.4% did not use a single dose of IPTP-SP. Based on the data below (Figure 1), (N = 165) used at least 3 doses of IPTP-SP which represents a prevalence of 43% for women who took three or more doses of IPTP-SP.

### 3.4 Individual-Level Factors Influencing the Uptake of +3 Doses of IPTP-SP

Bivariate logistic regression evaluated the association between individual factors and uptake of 3 or more IPTp-SP. Analysis showed an association between secondary education and uptake of 3 or more IPTp-SP (OR = 1.8, 95% CI: 1.20 – 2.78, p = 0.006). Starting the ANC clinic during the first trimester increased the odds of taking at least three doses of IPTp-SP drug by fourfold-fold (OR = 3.9, 95% CI: 2.39 – 6.34, p < 0.001), while those who started the ANC in their third trimester were 79% less likely to have taken the three doses (OR = 0.21, 95% CI: 0.12 – 0.37, p < 0.001). Those who had completed at least 4 ANC visits had almost a five-fold likelihood of taking at least 3 doses of IPTp-SP compared to those with fewer than four visits (OR = 4.9, 95% CI: 3.04 – 7.81, p < 0.001). Those earning over KES 10,000 having a close to threefold odds of uptake of 3 or more IPTp-SP drugs (OR = 2.7, 95% CI: 1.46 – 5.16, p = 0.002). The odds of taking three doses increased twofold among the participants who confirmed to be aware of IPTp-SP drug (OR = 2.5, 95% CI: 1.18 – 3.22, p = 0.04) and increased fivefold-fold for those who knew some benefits of IPTp-SP (OR = 5.5, 95% CI: 2.41 – 12.61, p < 0.001). Those who believed that attending ANC visits influenced their compliance with SP intake had a double chance of having taken 3 doses or more (OR = 2.1, 95% CI: 1.187 – 3.678, p = 0.011). The remaining individual factors showed statistically in significance association (Table 3).

A multivariable logistic regression was run for all the factors with statistically significant crude odds ratios (ORs) to identify the adjusted odds ratio (aOR), results as shown in Table 4 below. Starting ANC visits in the first trimester doubled the odds (aOR = 2.1, 95% CI: 1.23 – 3.67, p = 0.046), while making over 4 ANC visits increased the odds of taking at least three doses by three times (aOR = 3.1, 95% CI: 1.49 – 6.50, p = 0.002). A higher income increased the likelihood of a mother taking at least three doses of SP by two and a half times (aOR = 2.6, 95% CI: 1.24 – 5.36, p = 0.012). Being aware of the benefits of IPTp-SP drugs improved the chances of SP regime compliance by close to four times (aOR = 3.7, 95% CI: 1.40 – 9.74, p = 0.008). Those who believed ANC services were beneficial had a three times better chance of taking 3 or more SP doses (aOR = 3.2, 95% CI: 1.61 – 6.31, p = 0.001).

## 4 Discussion

### 4.1 The Prevalence of Uptake of Three or More Doses of IPTp-SP

The current study has shown that the prevalence of those who took 3 or more doses was 43%. This prevalence rate is comparable to that reported in previous studies. Pons-Duran *et al*. [10] reported that the prevalence at the national level to be less than 25% in DRC, Madagarscar, and Nigeria but at 63% in Mozambique. In Uganda, Martin *et al*. [11] reported a prevalence of 22.3%. In Kenya, Karoki *et al*. [12] reported a prevalence of 49%, although stating that it was lower in the lake region than in the coastal region. Arguably, the rates showed lower prevalence in comparison to the previous study performed in Kenya, with a possible reason for the difference. Karoki *et al*. [12] showed that the malaria prevalence rates in the lake region were lower than those from the coastal area, which may explain the differences between the overall endemic regions in the Kenyan prevalence of 49% compared to our rate of 43%. The findings offer a more appropriate estimate of the SP regime compliance rate in the region. These findings indicate noncompliance with WHO recommendations that every pregnant woman from malaria endemic regions should take at least three IPTp-SP drugs during the pregnancy period. The low prevalence could be attributed to individual-level factors as discussed below.

### 4.2 Individual-Level Factors Influencing IPTp-SP+3 Uptake

The study findings revealed that mothers with a higher monthly income had higher odds of uptake of 3 or more SP doses than those with lower income. Also, starting antenatal clinics as early as in the first trimester doubled the likelihood of a mother taking up at least three SP doses. In addition, making at least four ANC visits increases the possibility of taking at least three doses of SP. Furthermore, those with some knowledge of the benefits of IPTp-SP drugs and those who had a positive attitude toward ANC services were linked with better IPTp-SP uptake. These findings concur with previous studies. A study done in Sierra Leone showed that the rate of IPTp-SP uptake increased with increase in ANC visits [13]. Similarly, Azizi [14] evaluated compliance with at least 3 doses of SP in Malawi and reported that having at least 4 ANC visits was associated with high uptake of at least three doses of IPTp-SP. These findings are also supported by Mkalukwatage Mchwampaka *et al*. [15] in Tanzania and Ameyaw *et al*. [16] in Benin. These findings indicate that the higher number of visits the higher chances of mothers being sensitized about the drug, increasing its uptake.

The timing of the first ANC visit is correlated with a higher number of ANC visits. Consistent with our findings, Azizi *et al*. [17] showed that the number of dosages increased for women who had their first ANC clinic in the first trimester. Similar findings were also documented in a Ghanaian study by Amoako *et al*. [18], who found that women who began ANC visits in the first trimester were more than three times as likely to take three or more doses of SP than women who began in the second or third trimester. An intensive health education forum is often provided to mothers in their first ANC visit in interactive group sessions. This is followed by reinforced messaging during subsequent visits, explaining why most of those doing their first visits in the first trimester would record a higher IPTp-SP uptake.

The current study findings concur with Mkalukwatage Mchwampaka *et al*. [15] on the impact of awareness of IPTp-SP and its benefits in pregnancy toward enhancing uptake of at least three doses of the drug. Mkalukwatage Mchwampaka and colleagues showed a strong correlation between the uptake of three or more SP doses and knowing of IPTp-SP and having at least secondary education [15]. However, unlike the current study, Mkalukwatage Mchwampaka and colleagues showed that a higher parity had a statistically significant association with uptake of three or more doses of IPTp-SP [15]. Ideally, the knowledge of IPTp-SP could go hand in hand with the number of previous pregnancies since such repetitive sensitization enhanced knowledge as opposed to a mother who is visiting the facility for the first time. Additionally, higher education increases the ability to comprehend shared sensitization materials such as leaflets for the drugs, which may increase the active involvement with uptake of the drug.

According to Azizi *et al*. [17], employment increased the odds of taking at least 3 doses of SP by 2.5 times. These findings are related to the current studywhich indicate that higher monthly income improved uptake of the drug.

### 4.3 Limitations

The study was a cross-sectional study which is not ideal for drawing cause-effect associations due to antecedent-consequent bias common with this design. There was potential for bias in participant inclusion and selectiongiven that recruited mothers were only those that delivered past 37 weeks gestation, excluding a significant proportion of mothers and skewing data. Considering Busia County has an HIV prevalence of 6.7% [19], excluding HIV mothers could affectgeneralizability of study findings.

## 5 Conclusion

According to the study, less than half of the pregnant women in Busia County are taking the recommended three doses of IPTp-SP. Even though it is widely known that preventing malaria is crucial to addressing related complications during pregnancy, there are significant factors that hinder the proper absorption of the medication. This study has shown that individual-related factors associated with the uptake of at least three doses of the IPTp-SP drug include the time of the first ANC visit, the number of ANC visits, income level, mothers’ awareness of the IPTp-SP drug, and a positive attitude toward the significance of antenatal clinics. This study has shown the importance of optimizing the number of mothers accessing ANC services, engaging in sensitization for IPTp-SP drugs to the community and emphasizing drug benefits, access points, and dosages. Further research should target to include HIV-positive mothers on daily CTX to determine malaria prevention in pregnancy in the region and include mothers with premature deliveries and compare the outcome Vis a Vis the uptake of three or more doses of IPTp-SP.

## Figures and Tables

**Table 1:** Baseline sociodemographic characteristics.

Variable	Frequency (N/384)	Percentage
**Age**		
≤ 19 Yrs.	51	13.3%
20 – 25 Yrs.	129	33.6%
26 – 30 Yrs.	106	27.6%
31 – 35 Yrs.	60	15.6%
≥ 36 Yrs.	38	9.9%
*Mean age = 26.51 Standard Deviation = 6.1*		
**Education Level**		
No education	9	2.3%
Primary	148	38.5%
Secondary	163	42.4%
Tertiary	64	16.7%
**Marital Status**		
Single	76	19.8%
Married	307	79.9%
Separated/Widowed	1	0.3%
**Occupation**		
Unemployed	203	52.9%
Student	28	7.3%
Casual Laborer	38	9.9%
Self-employed	87	22.7%
Formal employment	28	7.3%
**Residency**		
Rural	354	92.2%
Urban	30	7.8%
**Monthly Income (Ksh.)**		
<10000	336	87.5%
10000 – 20000	46	12.0%
20001 – 50000	2	0.5%
50001 +	0	0%

**Table 2:** Basic obstetric characteristics.

Variable	Frequency (N = 386)	Percentage (100%)
**Parity**		
0	02	0.5%
1 - 2	238	**62.0%**
3 - 4	103	26.8%
5 plus	41	10.7%
**Gestation at 1 ANC Visit**		
1^st^ Trimester	98	25.5%
2^nd^ Trimester	195	**50.8%**
3^rd^ Trimester	91	23.7%
**Number of ANC Visits Done**		
0 visits	00	0.0%
1 visit	13	3.4%
2 Visits	50	13.0%
3 Visits	84	21.9%
4 Visits	103	**26.8%**
5 Visits	70	18.2%
>5 Visits	64	16.7%
**ANC Service Point**		
Mission	03	0.8%
Private	13	3.4%
Public	368	95.8%

**Table 3:** Bivariate Association for Individual Factors with IPTp-SP Uptake.

Variable	≥ 3 IPTp-SP Drugs Uptake *N = 165*	<3 IPTp-SP Drug Uptake *N = 219*	Crude Odds Ratio (95% CI)	P-value
Age				
*≤ 25 Yrs.*	79 (47.9%)	101 (46.1%)	1.07	0.757
*≥ 26 Yrs.*	86 (52.1%)	118 (53.9%)	(0.716 – 1.609)	
Education Level				
*Secondary Plus*	111(67.3%)	116 (53.0%)	**1.83**	**0.006**
*Primary or None*	54 (32.7%)	103 (47.0%)	(1.200 – 2.777)	
Marital Status				
*Married*	132 (80.0%)	175 (79.9%)	1.00	0.544
*Single*	33 (20.0%)	44 (20.1%)	(0.607 – 1.666)	
Employment Status				
*Employed*	67 (40.6%)	86 (39.3%)	1.06	0.833
*Unemployed*	98 (59.4%)	133 (60.7%)	(0.700 – 1.597)	
Parity				
Para 2 or less	106 (64.2%)	134 (61.2%)	1.14	0.595
Para 3 or more	59 (35.8%)	85 (38.8%)	(0.750 – 1.732)	
First ANC				
First Trimester	66 (40.0%)	32 (14.6%)	**3.90**	**<0.001**
Other Trimesters	99 (60.0%)	187 (85.4%)	(2.393 – 6.343)	
First ANC				
Second Trimester	83 (50.3)	112 (51.1%)	0.97	0.918
Other Trimesters	82 (49.7%)	107 (48.9%)	(0.646 – 1.449)	
First ANC				
Third Trimester	16 (9.7%)	75 (34.2%)	**0.21**	**<0.001**
Other Trimesters	149 (90.3%)	144 (65.8%)	(0.115 – 0.371)	
Number of ANC Visits				
≥ 4 Visits	134 (81.2%)	103 (47.0%)	**4.87**	<0 001
Below 4 Visits	31 (18.8%)	116 (53.0%)	(3.036 – 7.806)	
ANC Facility				
Public Hosp.	156 (94.5%)	212 (96.8%)	0.57	0.309
Private Hosp.	9 (5.5%)	7 (3.2%)	(0.209 – 1.570)	
Level of Income				
Over Ksh. 10,000	31 (18.8%)	17 (7.8%)	**2.74**	**0.002**
Below Ksh. 10,000	134 (81.2%)	202 (92.2%)	(1.463 – 5.164)	
Aware of Malaria signs				
Yes	157 (96.3%)	206(94.5%)	1.52	0.472
No	6 (3.7%)	12 (5.5%)	(0.241 – 2.832)	
Aware of IPTp-SP				
Yes	132 (80.0%)	135 (61.6%)	**2.48**	**0.040**
No	33 (20.0%)	84 (38.4%)	(1.180 – 3.218)	
Educated on Malaria at ANC				
Yes	131 (79.4%)	166 (75.8%)	1.23	0.460
No	34 (20.6%)	53 (24.2%)	(0.896 – 3.180)	
Felt ANC Visits Tiresome				
Yes	25 (15.2%)	58 (26.5%)	0.50	0.080
No	140 (84.8%)	161 (73.5%)	(0.294 – 1.834)	
Occupation Limits ANC				
Yes	21 (12.7%)	24 (11.0%)	1.18	0.632
No	144 (87.3%)	195 (89.0%)	(0.635 – 2.211)	
IPTp Benefits ↑ uptake				
Yes	158 (95.8%)	176 (80.4%)	**5.52**	**<0.001**
No	7 (4.2%)	43 (19.6%)	(2.411 – 12.611)	
Positive Attitude on ANC Services				
Yes	145 (87.9%)	170 (77.6%)	**2.09**	**0.011**
No	20 (12.1%)	49 (22.4%)	(1.187 – 3.678)	
Source of Malaria information				
Hospital	122 (73.9%)	143 (65.9%)	1.17	0.310
Others	43 (26.1%)	74 (34.1%)	(0.73 – 18.93)	

**Table 4:** Multivariate Association for Individual Factors and significant variables from the bivariate association.

Variable	Crude Odds Ratio (95% CI)	P-value	Adjusted Odds Ratio (95% CI)	P-value
Education Level				
*Secondary Plus*	1.83	**0.006**	1.3	0.310
*Primary or None (Ref)*	(1.200 – 2.777)		(0.785 – 2.141)	
1^st^ ANC at First Trimester				
Yes	3.90	**<0.001**	2.12	**0.046**
No (*Ref)*	(2.393 – 6.343)		(1.229 – 3.669)	
1^st^ ANC at Third Trimester				
Yes	0.21	**<0.001**	0.67	0.377
No (*Ref)*	(0.115 – 0.371)		(0.278 – 1.624)	
Number of ANC Visits				
4 or more	4.87	**<0.001**	3.12	**0.002**
Less than 4 *(Ref)*	(3.036 – 7.806)		(1.494 – 6.500)	
Income				
Over Ksh. 10000	2.74	**0.002**	2.58	**0.012**
Below Ksh. 10000 *(Ref)*	(1.463 – 5.164)		(1.236 – 5.361)	
Aware of IPTp-SP				
Yes	2.48	**0.040**	0.69	0.174
No (*Ref)*	(1.180 – 3.218)		(0.400 – 1.181)	
IPTp-SP Benefits ↑ uptake				
Yes	5.52	**<0.001**	3.69	**0.008**
No (*Ref)*	(2.411 – 12.611)		(1.396 – 9.744)	
Positive Attitude on ANC Services				
Yes	2.09	**0.011**	3.18	**0.001**
No (*Ref)*	(1.187 – 3.678)		(1.605 – 6.311)	

## Data Availability

All relevant data pertaining to this study are available on the manuscript. The raw data sets are available with the Principal Investigator upon request.

## List of Abbreviations

ANC: Antenatal care
aOR: Adjusted odds ratio
CI: Confidence interval
IPTp: Intermittent preventive treatment during pregnancy with sulfadoxine-pyrimethamine
KMIS: Kenya Malaria Indicator Survey
LBW: Low birth weight
MiP: Malaria in pregnancy
OR: Odds ratio
SDGs: Sustainable Development Goals
WHO: World Health Organization

## References

[1] World Health Organization (2021). World Malaria Report 2021.

[2] (2015). Kenya Malaria Indicator Survey.

[3] Wanyonyi WA, Mulambalah CS, Mulama DH, Omukunda E (2019). Malaria Prevalence and Risk Analysis among Pregnant Women in Bungoma County, Kenya. Medical Sciences.

[4] Guyatt HL, Snow RW (2001). Malaria in Pregnancy as an Indirect Cause of Infant Mortality in Sub-Saharan Africa. Transactions of the Royal Society of Tropical Medicine and Hygiene.

[5] WHO (2019). Intermittent Preventative Treatment to Reduce the Risk of Malaria during Pregnancy.

[6] Nyamu GW, Kihara JH, Oyugi EO, Omballa V, El-Busaidy H, Jeza VT (2020). Prevalence and Risk Factors Associated with Asymptomatic *Plasmodium falciparum* Infection and Anemia among Pregnant Women at the First Antenatal Care Visit: A Hospital Based Cross-Sectional Study in Kwale County, Kenya. PLOS ONE.

[7] (2020). Kenya Malaria Indicator Survey.

[8] Busia County (2022). County Government of Busia County Integrated Development Plan 2018-2022 II Busia County Vision.

[9] Israel GD (1992). Determining Sample Size 1. 1-5.

[10] Pons-Duran C, Llach M, Sacoor C, Sanz S, Macete E, Arikpo I (2021). Coverage of Intermittent Preventive Treatment of Malaria in Pregnancy in Four Sub-Saharan Countries: Findings from Household Surveys. International Journal of Epidemiology.

[11] Martin MK, Venantius KB, Patricia N, Bernard K, Keith B, Allen K (2020). Correlates of Uptake of Optimal Doses of Sulfadoxine-Pyrimethamine for Prevention of Malaria during Pregnancy in East-Central Uganda. Malaria Journal.

[12] Karoki SM, Kariuki L, Owiti PO, Takarinda KC, Kizito W, Edwards JK (2016). Intermittent Preventive Treatment and Bed Nets Uptake among Pregnant Women in Kenya. East African Medical Journal.

[13] Buh A, Kota K, Bishwajit G, Yaya S (2019). Prevalence and Associated Factors of Taking Intermittent Preventive Treatment in Pregnancy in Sierra Leone. Tropical Medicine and Infectious Disease.

[14] Azizi SC (2020). Uptake of Intermittent Preventive Treatment for Malaria during Pregnancy with Sulphadoxine-Pyrimethamine in Malawi after Adoption of Updated World Health Organization Policy: An Analysis of Demographic and Health Survey 2015-2016. BMC Public Health.

[15] Mchwampaka WM, Tarimo D, Chacky F, Mohamed A, Kishimba R, Samwel A (2019). Factors Affecting Uptake of ≥ 3 Doses of Sulfadoxine-Pyrime-thamine for Malaria Prevention in Pregnancy in Selected Health Facilities, Arusha Region, Tanzania. BMC Pregnancy and Childbirth.

[16] Ameyaw EK, Njue C, Amoah RM, Appiah F, Baatiema L, Ahinkorah BO (2021). Is Improvement in Indicators of Women’s Empowerment Associated with Uptake of WHO Recommended IPTp-SP Levels in Sub-Saharan Africa? A Multilevel Approach. BMJ Open.

[17] Azizi SC, Chongwe G, Chipukuma H, Jacobs C, Zgambo J, Michelo C (2018). Uptake of Intermittent Preventive Treatment for Malaria during Pregnancy with Sulphadoxine-Pyrimethamine (IPTp-SP) among Postpartum Women in Zomba District, Malawi: A Cross-Sectional Study. BMC Pregnancy Childbirth.

[18] Amoako BK, Anto F (2021). Late ANC Initiation and Factors Associated with Sub-Optimal Uptake of Sulphadoxine-Pyrimethamine in Pregnancy: A Preliminary Study in Cape Coast Metropolis, Ghana. BMC Pregnancy Childbirth.

[19] Kenya AIDS Strategic Framework II.

